# Algorithm of genetic diagnosis for patients with head and neck paraganglioma—update

**DOI:** 10.3389/fneur.2024.1437027

**Published:** 2024-08-29

**Authors:** Katarzyna Radomska, Zofia Leszczyńska, Rafal Becht, Monika Zaborek - Łyczba, Anna Rzepakowska, Jakub Lubiński, Marcin Szymański

**Affiliations:** ^1^Department of Otolaryngology, Pomeranian Medical University, Szczecin, Poland; ^2^Department of Oncology, Pomeranian Medical University, Szczecin, Poland; ^3^Department of Otolaryngology, Medical University of Lublin, Lublin, Poland; ^4^Department of Otolaryngology, Medical University of Warsaw, Warsaw, Poland

**Keywords:** head and neck, tumor, paraganglioma, pheochromoacytoma, geneti algorithm, genetic diagnosis

## Abstract

Paragangliomas are rare tumors originating from the paraventricular bodies of the autonomic nervous system located in the adrenal glands, chest, abdomen, pelvis and head and neck. Tumors of this type account for 0.5% of head and neck cancers, 0.03% of all cancers and their incidence is estimated at 1–30/100,000 per year. Head and Neck Paragangliomas (HNPGL) are localized in carotid body, tympanic cavity or jugular foramen. It is established that HNPGL may be associated with mutations of the SDH complex, with SDHD being the most prevalent. However, SDHB, SDHC and SDHAF are also potential causes. The aforementioned mutations are influenced by various risk factors, including young age, a positive family history of paraganglioma, the presence of metastases and gender The purpose of this study is to summarize the results of genetic testing performed on patients with head and neck paraganglioma and to create an up-to-date genetic diagnosis algorithm for patients with HNPGL based on previous studies published in the literature that can be used in daily practice. Several papers observed that among SDHD mutation carriers, most or all of those studied had HNPGL, and SDHB mutations were more frequently found in the presence of metastasis. Based on the results, it was concluded that there is no basis for genetic testing for VHL in patients without a positive family history. In each algorithm proposed by different authors, proposals for rational genetic diagnosis were analyzed based on the studies cited by the author and the analyses included in our paper. For the analysis of the treatment algorithms, the following were included: Martin, Mannelli, Neumann, Gupta. Subsequently, publications related to the genetic diagnosis of HNPGL were analyzed to verify the proposed algorithms in light of the latest genetic studies and to establish an updated diagnostic management scheme.

## Introduction

Paragangliomas are rare tumors originating from the paraventricular bodies of the autonomic nervous system (1) located in the adrenal glands, chest, abdomen, pelvis and head and neck. Tumors of this type account for 0.5% of head and neck cancers, 0.03% of all cancers (2), and their incidence is estimated at 1–30/100,000 per year (3). Paragangliomas can originate from the paraganglial bodies of the sympathetic and parasympathetic nervous system, with head and neck paragangliomas (HNPGLs) originating from parasympathetic bodies and are not catecholamine-secreting tumors (1, 2, 4–8). Sympathetic chain PGLs more commonly secrete catecholamines compared to other PGLs, though all HNPGLs may potentially possess this feature (9). A recent study reports that only 3.7% of all HNPGLs lead to elevated normetanephrine levels. Interestingly, this study also found higher levels of methoxytyramine, the O-methylated metabolite of dopamine in benign HNPGLs, which contrasts the prior suggestion of an increase in methoxytyramine and dopamine in metastatic PGLs (10). The tumors are most often benign, but 10–15% may metastasize to bone, liver, lung or lymph nodes (1, 11–13). The current World Health Organization classification divides these tumors into non-metastatic and metastatic (13–15) and recommends using this term instead of “malignant tumors.” They can occur as sporadic, multiple or as part of genetically determined syndromes (3, 7, 16–18).

Genetic testing in patients with paragangliomas is complementary to diagnosis and allows management to be targeted to detect potential multiple tumors or metastases. In addition, tests performed in family members can lead to the diagnosis of paraganglioma at an early stage, while they are still asymptomatic, and implement appropriate management. It is established that HNPGL may be associated with mutations of the SDH complex, with SDHD being the most prevalent. However, SDHB, SDHC and SDHAF are also potential causes. The aforementioned mutations are influenced by various risk factors, including young age, a positive family history of paraganglioma, the presence of metastases and gender (1, 3, 5, 12).

To date, no such work has been conducted for the Polish population, and in determining the indications for diagnosis it is necessary to rely on the results of studies for groups of patients from other countries. Nevertheless, at present, the extension of diagnostics in patients with HNPGL by genetic testing should be an essential part of the treatment process. To this end, bearing in mind also the potential costs of testing, it is worth using a diagnostic algorithm.

## Aim of the study

The purpose of this study is to summarize the results of genetic testing performed on patients with head and neck paraganglioma and to create an up-to-date genetic diagnosis algorithm for patients with HNPGL based on previous studies published in the literature that can be used in daily practice.

## Methodics

Databases from Medline, Embase, Scopus, Google Scholar from 2000 to X 2023 were searched. Inclusion criteria included papers describing genetic findings in patients with head and neck paragangliomas. Original papers, review papers, and case reports and family studies describing a genetic background or family history of HNPGL were included in the review. Search words used—head and neck paraganglioma (HNPGL) genetic spectrum, HNPGL genetic classification, hereditary HNPGL, SDH mutations in HNPGL, genetic overview of HNPGL, genetic testing in HNPGL. We searched for all results related to the genetic background of HNPGL as well as included review papers that proposed algorithms for genetic diagnosis. Papers examining paragangliomas outside the head and neck region were excluded from the analysis. Also, analyses investigating the molecular mechanisms of mutation effects on PGL development, without clinical analysis, were not included in this paper. Due to the rarity of HNPGL, case reports and family line studies were included in the paper.

Data were downloaded on genetic test results and their correlation with clinical data. The genetic diagnostic algorithms proposed so far were analyzed. Algorithms related to diagnostic imaging or proposed treatment were not included in the study.

**Figure 1:**
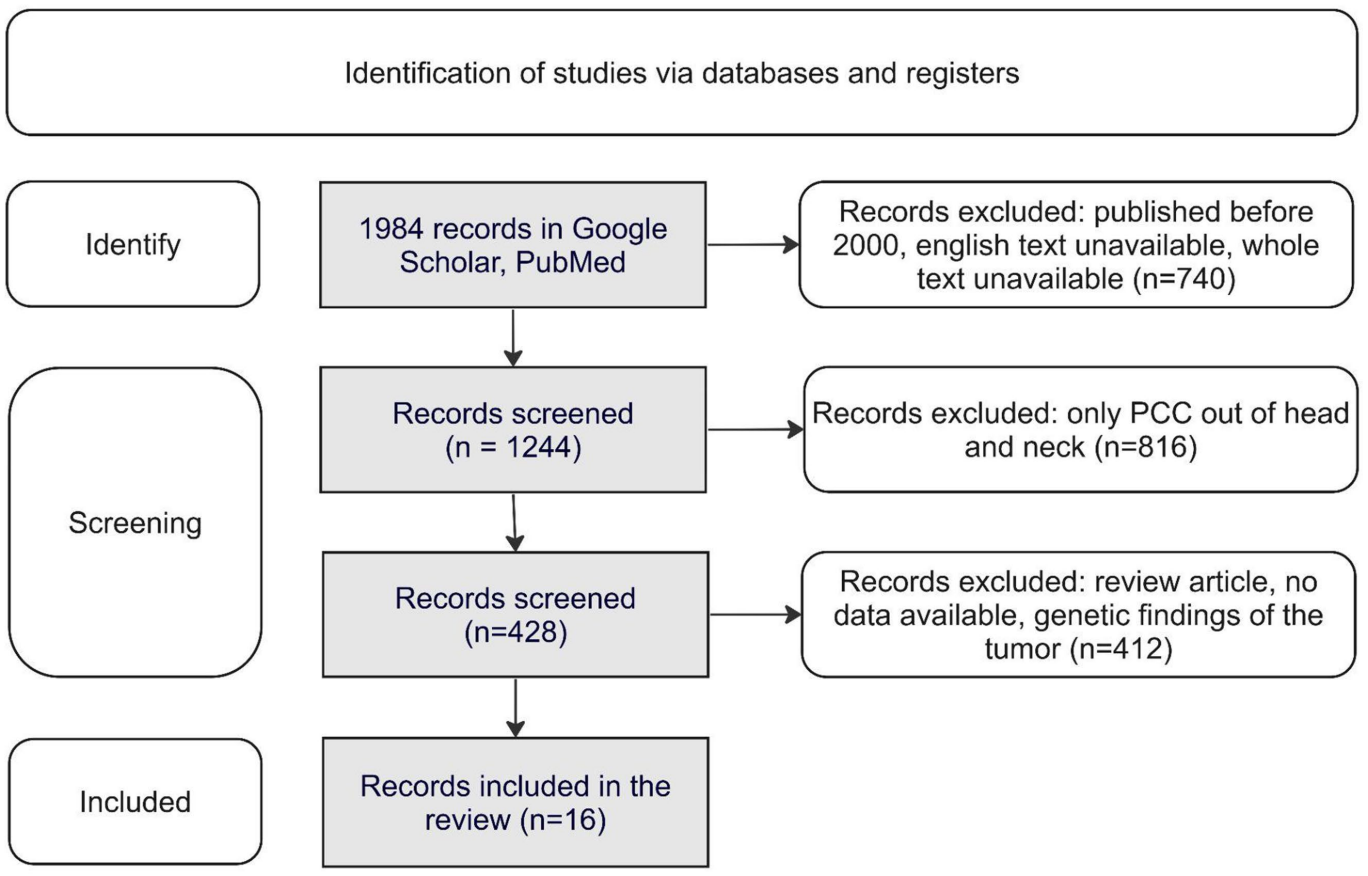
Identification of studies via databases and registers. PCC, Pheochromocytoma.

The selection of publications for the study is shown in Figure 1.

## Results

The results of genetic testing for HNPGL are shown in Table 1, and papers with repeated patient groups are omitted. The most common mutations found were in the SDH complex (2, 22, 25, 27, 28), with mutations within SDHD most common for HNPGL, and SDHB, SDHC and SDHAF2 mutations less common. The papers showed that the main risk factors for mutations were young age, a positive family history of PCC/PGL, multifocality (12, 20, 24), metastatic tumors (lymph nodes, bone, liver, lung) and gender (22). However, there remained a group of patients without risk factors who also had genetic mutations (17).

**Table 1:** Genetic testing in patients with HNPGL in order of publication years.

	Number of patients with HNPGL	Mutations	Mutation risk factors
Boedecker 2007 (5)	195 patients HNPGL	SDHB, SDHD, SDHC	Mutations were found in 53.8% of patients with HNPGL. Earlier onset of disease compared to those with sporadic HNPGL. Transmission from the father increases the risk of HNPGL, from the mother the risk as in the general population.
Burchinon 2009 (12)	445 HNPGL i/lub thoracic-abdominal or pelvic PGL, w tym 330 z HNPGL	SDHB, SDHC, SDHD	Mutation rate: 55% in the whole group, 40% of patients with sporadic tumors, 49 out of 99 with single cervical PGL, 35% with single cervical PGL, 0% with isolated tympanic PGL lower age in patients with mutations, higher number of patients under 35 years of age in patients with mutations, multiple tumors and positive family history significantly more common in those with mutations,
Mannelli 2009 (19)	501 patients PCC/HNPGL 105 z HNPGL 24 multifocal, 81 single	wild type RET, SDHB, SDHC, SDHD	SDHB—most associated with malignancy 33% of patients with HNPGL had mutation in SDH group, only 2 patients with positive family history of VHL, most often SDHD (26.4%), SDHB (3.8%), SDHC (2.8%) Single tumors, negative family history of HNPGL 14.3% with mutations, 82% of germline mutations detected in patients under 50 years of age
Neumann 2009 (20)	598 patients from different countries, the largest study involving only HNPGL,	SDHD, SDHC, SDHB	183 (30.6%) patients—germline mutation in SDHx gene, frequency (decreasing): SDHD, SDHB, SDHC Six factors were found to suggest the presence of a germline mutation: age (<40 years), gender, number of tumors (single or multiple), tumor biology (malignant or benign HNP), previous chaperone tumors and family history of HNPGL or pheochromocytoma
Bayley 2010 (21)	443 patients with seemingly sporadic PCC/PGL	Tested SDHAF2	SDHAF2 mutation testing is warranted for young patients with HNPGL if no mutations in SDHD, SDHB, and SDHC are found
Kunst 2011 (4)	57 related persons, including 23 carriers	SDHAF2 (PLG2-SDH5)	SDHAF2—early onset of disease and high risk of multifocal, some patients were asymptomatic, tumors were detected through family coverage, early onset—diagnosis at an average age of 33. Patients inheriting mutation from mother, do not have PGL disease (“cancer free carriers”)
Piccini 2012 (17)	79 patients with HNPGL 114 tumors (33 patients previously described in Mannelli 2009)	SDHA, SDHB, SDHC, SDHD, SDHAF2, VHL, MAX, and TMEM127	Division into 3 groups: Positive family history—all—had SDHD mutations. Multiple HNPGL, family history negative—all—were carriers of SDHD Without risk factors (SDHD, VHL, SDHB, SDHAF)—18.8% carried germline mutations 16 cases of eardrum tumors, 5 showed mutations of which 3 were isolated tumors and 3 had other HNPGL.—The only study to show mutations in isolated tympanic tumors most frequently—SDHD, less frequently SDHB, SDHC and SDHAF2
Chen 2017 (22)	37 patients with HNPGL	SDH mutations and 10 suppressor genes	SDH mutations—faster progression, faster tumor onset, multifocal lesions and malignancy; SDH mutations have been shown to be associated with the number of methylated suppressor genes.
Heesterman 2018 (23)	222 mutation carriers SDHD	SDHD	During the 22-year follow-up period, 73% of patients developed another HNPGL. Men developed new cancer more often than women. The risk of new lesions decreases with the number of HNPGL at the time of first diagnosis. Just under 50% reported new symptoms. New cranial nerve palsies in 11%. During follow-up (average 7 years), 34% developed a new HNPGL; after 2 years, 7% did.
Ding 2019 (24)	23 HNPGL 9 M i 14 K, age 25–69	SDHD, 2 New mutatins—c.387_393del7 w SDHD i c.3247A > G w RET	Mutation was more common in patients with multifocal HNPGL Family 1: 12 individuals out of 32 family members tested had mutations in SDHD Family 2: 3 cases of SDHD mutations Family 3: 2 cases of SDHD gene mutations 10 patients with sporadic tumors had five cases of SDHD gene mutations and one case of RET gene mutations.
Riijken 2019 (18)	147 patients with HNPGL, genetic testing available for 98 patients	SDHD (65%) i SDHB (10%)	SDHB—higher mortality compared to SDHD SDHB—more frequent metastasis and worse prognosis SDHD mutations in 78% of patients with positive family history, mutation risk factors: positive family history, multifocal tumors
Bayley 2020 (25)	950 patients (PPGL or HNPGL),	germinal in genes encoding SDHB, SDHC lub SDHD	HNPGL risk (greatest to least): SDHD truncating and missense, SDHB missense, SDHB truncating. SDHB less frequent in HNPGL, more frequent in PPGL. SDHB and SDHD in slightly younger PPGL patients. In this study, the PPGL and HNPGL groups were considered together.
Bayley 2023 (26)	448 (HNPGL i PPGL)	SDHD, SDHB	SDHD truncating increases risk of PPGL and metastasis SDHB truncating increases the risk of PPGL and malignancy by 20%

Several papers observed that among SDHD mutation carriers, most or all of those studied had HNPGL (8, 12, 18, 19, 22, 29, 30), and SDHB mutations were more frequently found in the presence of metastasis (12, 18, 19, 22). Based on the results, it was concluded that there is no basis for genetic testing for VHL in patients without a positive family history (12, 31).

In each algorithm proposed by different authors, proposals for rational genetic diagnosis were analyzed based on the studies cited by the author and the analyses included in our paper. For the analysis of the treatment algorithms, the following were included: Martin (32), Mannelli (19), Neumann (20), and Gupta (31). Subsequently, publications related to the genetic diagnosis of HNPGL were analyzed to verify the proposed algorithms in light of the latest genetic studies and to establish an updated diagnostic management scheme.

**Figure 2:**
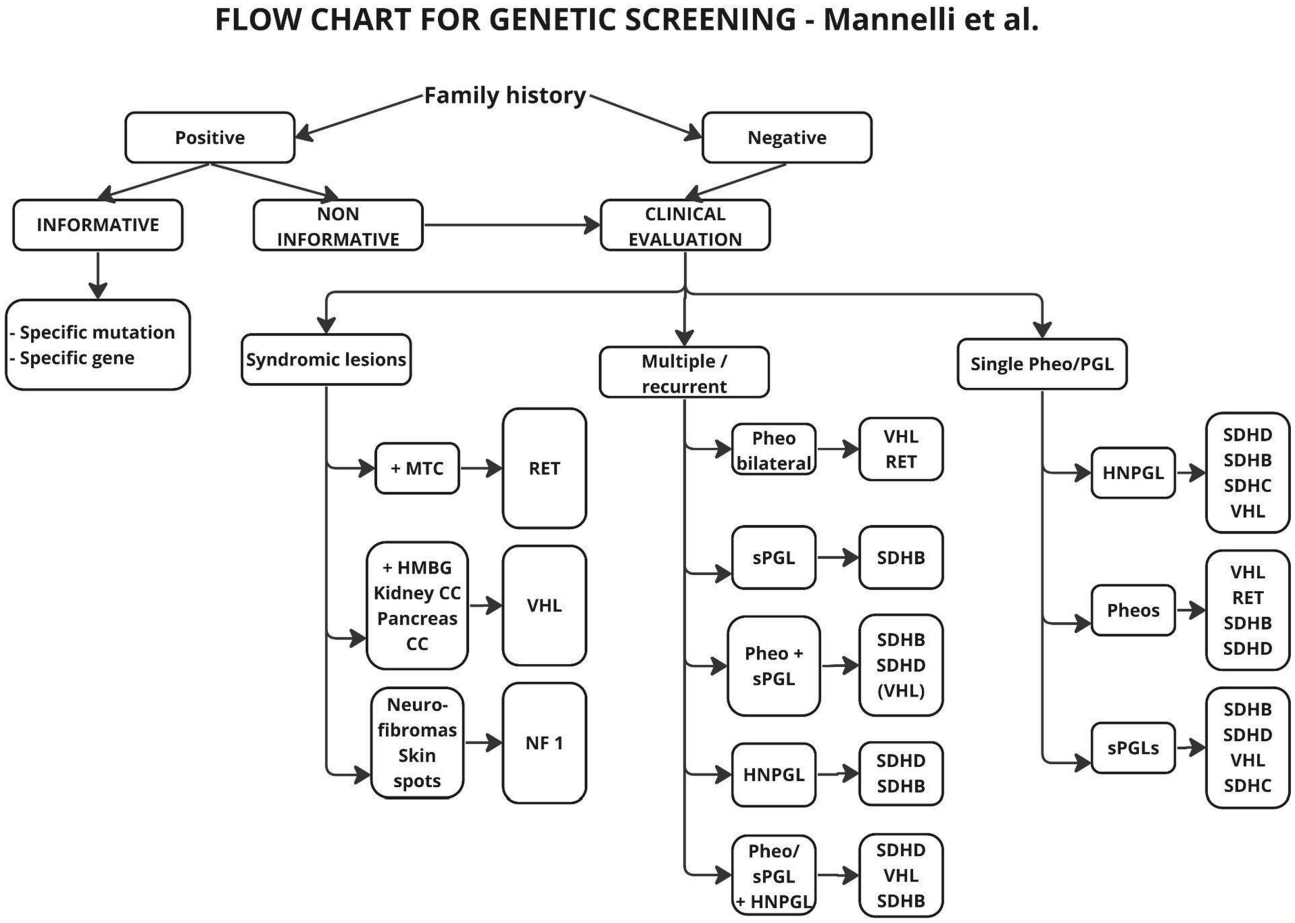
Flow chart suggested for genetic analysis in patients affected by Pheos or PGLs. The genes reported in the boxes are those more likely to be found mutated according to clinical picture. MTC, Medullary thyroid carcinoma; HMGB, hemangioblastomas; CC, cancer/cysts.

The first of these algorithms, proposed by Martin et al. (32), deals with the diagnosis of paragangliomas in general, without dividing them into HNPGL or other tumors, and only proposes genetic diagnosis with SDHD, SDHB and SDHC testing in one aspect, without considering possible risk factors. With the development of genetic testing, new and different schemes have emerged, making diagnosis more targeted, depending on the primary location and the presence of additional factors (19) (Figure 2).

**Figure 3:**
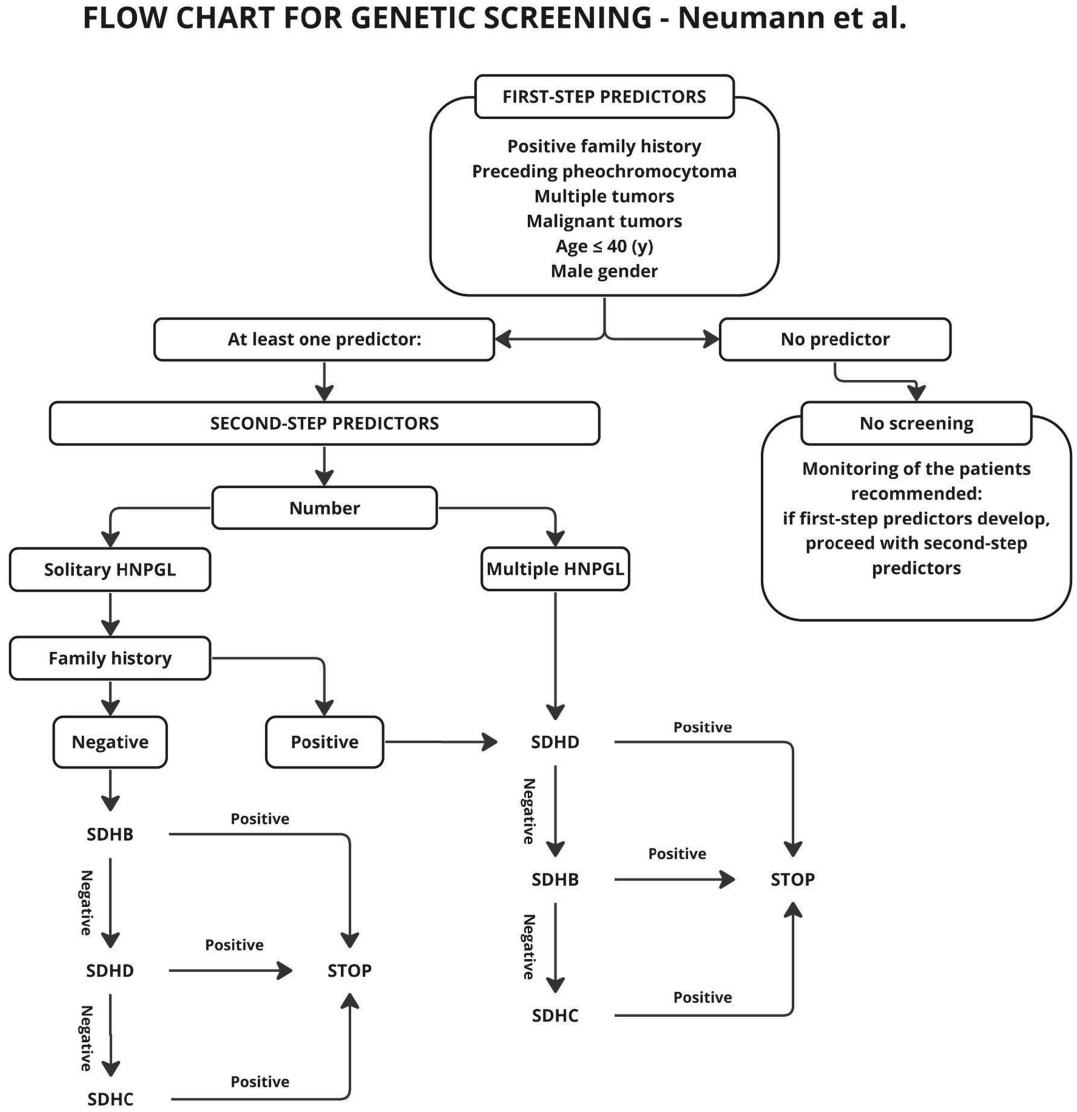
General algorithm for mutation screening of HNP patients. Only those with a first-step predictor being positive enter the main algorithm where second-step predictors are applied.

In the above scheme, a positive family history and multifocal tumor were taken as risk factors for mutations, but the age of the patients was not taken into account. In the description included in the paper, it is suggested in the aspect of age, to take 50 years as a cut-off point and above this age to abandon genetic testing, since only 5% of patients in this group showed mutations. Other authors (20, 31) suggest 40 years or even 35 years as the cutoff age (12, 20) (Figure 3).

**Figure 4:**
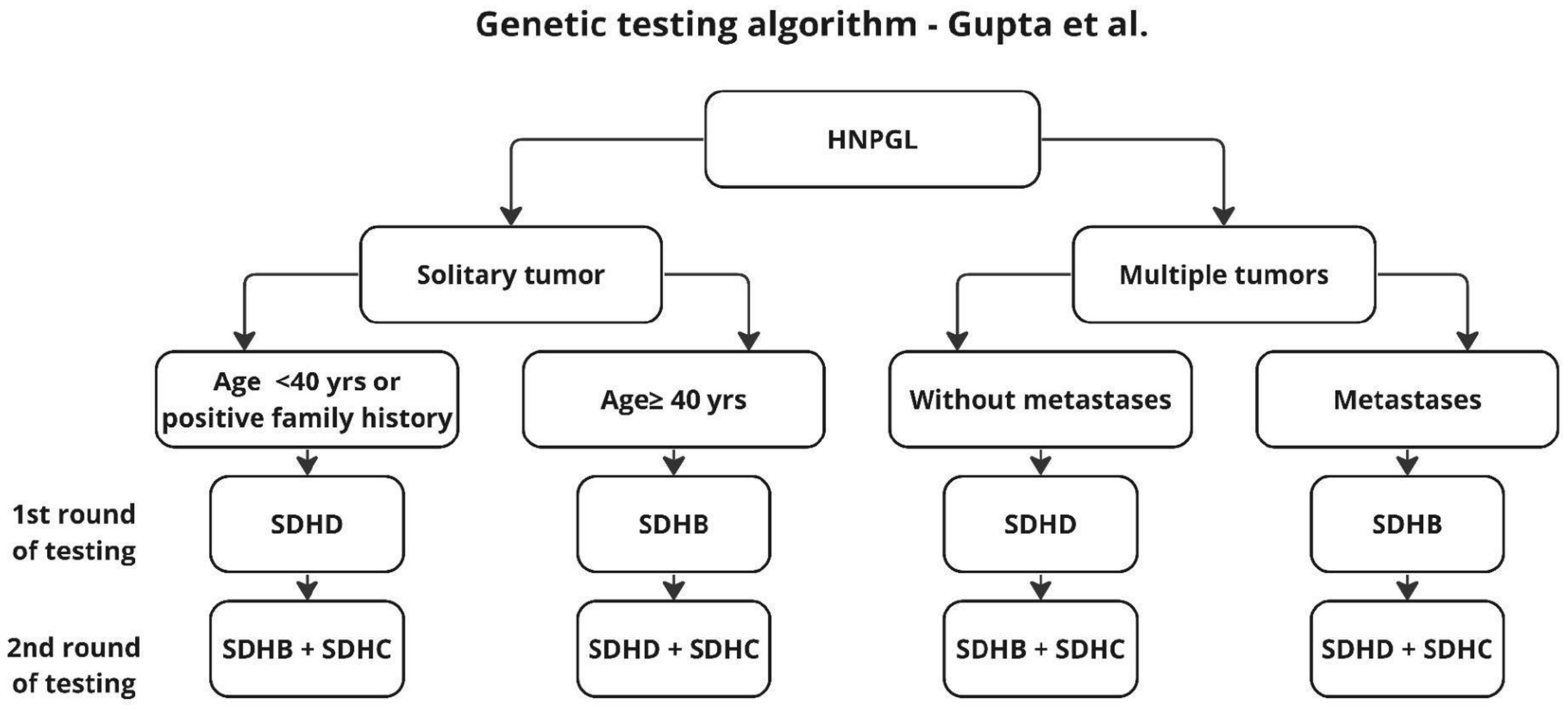
Algorithm according to Gupta et al. (31).

In the scheme proposed by Neumann et al. (20), genetic testing was to be performed only in patients with risk factors such as positive family history, previous pheochromocytoma, multiple tumors, malignant tumors, age under 40 years or gender. As shown in later studies, also patients without the aforementioned risk factors may carry genetic mutations, and genetic diagnosis should be performed for this group as well (31, 33). The publication of subsequent studies resulted in the algorithm described by Gupta et al. (31), shown in Figure 4.

**Figure 5:**
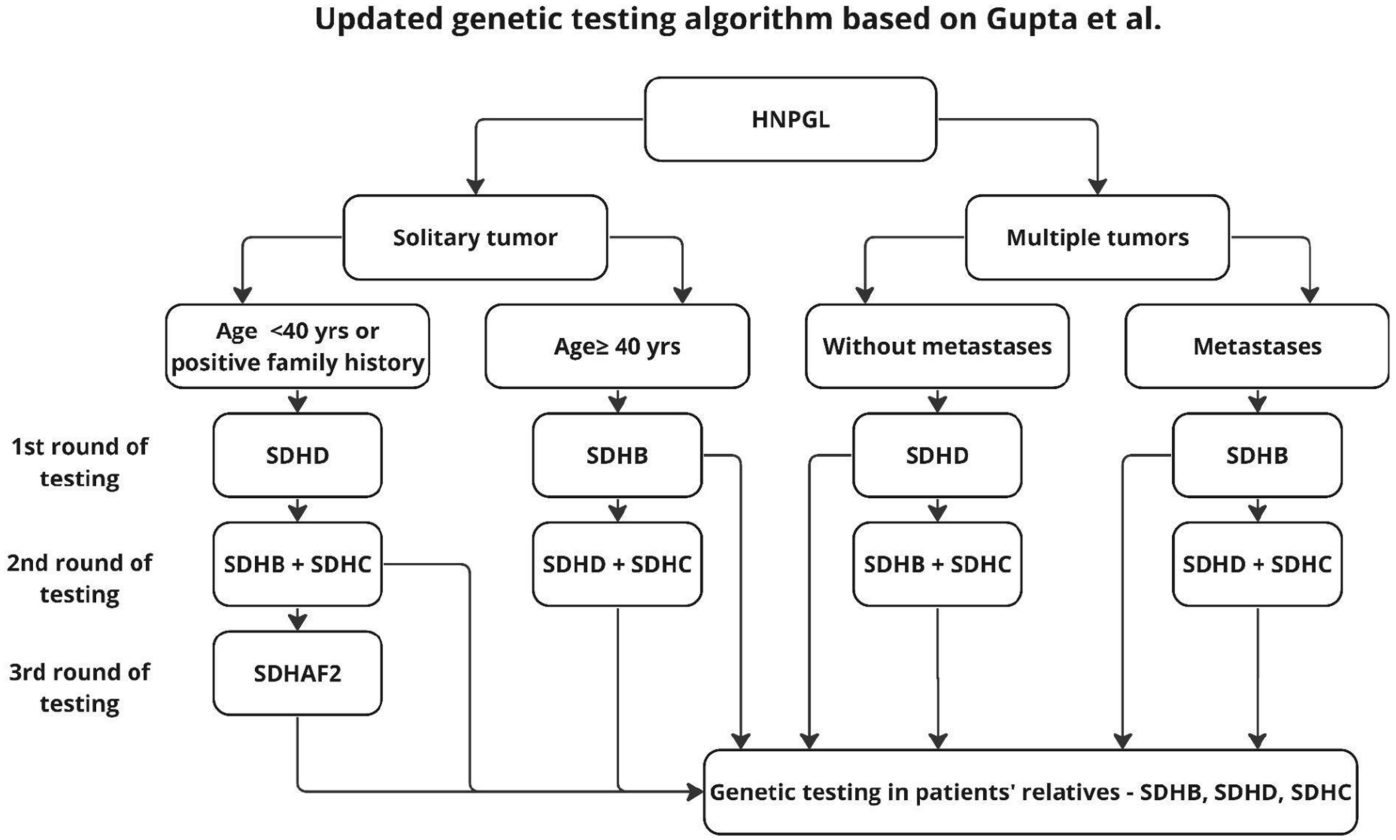
Updated algorithm based on Gupta’s proposal.

The algorithm proposed by Gupta applies only to HNPGL, dates back to 2019, and proposes two stages of testing to reduce diagnostic costs. In the first stage, the authors recommend genetic testing according to the risk factors found and an algorithm for patients without such factors, with the firąst stage additionally distinguishing age groups, unlike previous authors who proposed genetic testing for the entire SDHx group (19, 20, 32). This approach seems reasonable because, as shown in earlier work, 2 mutations are not found in the same patient (20). However, in light of the results of other studies, it seems reasonable to supplement the above algorithm with SDHAF2 testing, in patients younger than 40 years and in multifocal tumors (4, 21) (Figure 5).

## Discussion

Over the past few years, one can observe changes related to the approach to genetic diagnosis in paragangliomas. Undoubtedly, this is related to the rapid expansion of knowledge about different types of mutations and their impact on tumor biology. Not insignificant is the fact of developing diagnostic methods and improving accessibility to such tests. Based on the data collected from the literature, it can be noted that initially genetic testing was recommended for patients with HNPGL only in groups with risk factors, i.e., patients with young age, positive family history of pheochromocytoma, in multiple, metastatic tumors (formerly called malignant). As research has evolved, some recommendations have evolved or new ones have emerged.

Young age for different authors did not always mean the same metric age. Depending on the author, it ranged from 35 to 50 years of age (12, 19, 20, 22, 34). Burnichon et al. determined a young age for patients <35 years old, with SDHD, SDHB and SDHC mutations (12). Mannelli et al. did not recommend genetic testing in patients >50 years of age, justifying this with the observation that 82% of patients with mutations were younger than 50 (19). After Piccini extended the study in 2012, age was lowered to 45 years—as a risk factor for mutations (17). In a group of 598 patients with HNPGL described by Neumann et al. (20), mutations were found significantly more often for a population aged <40 years.

In the following years, studies were conducted on additional groups of genes which allowed the detection of SDHAF2 mutations (21) in young patients and recommended these tests if previous tests for SDHD, SDHB and SDHC are negative. These observations were confirmed in Kunst’s study, where patients with SDHAF2 mutation were found to have PGL in patients up to 33 years of age after further diagnostic imaging. The study by Zhu et al. 2015 (34)—studied 23 patients with HNPGL and a negative family history. Showed mutations in 8 patients, of which 7 occurred in patients younger than 45 years of age. When this group of patients was included in the larger Chen study (22), <40 years of age was considered young.

In terms of positive family history, the findings are consistent and confirm a higher frequency of mutations in these groups (12, 17–20, 24). There is little variation in mutation frequency from 78% (18) to 100% (17) for this group of patients.

In multiple, multifocal tumors, mutations are found with a frequency ranging from 38.8% (19), to as high as 100% if HNPGL coexists with PCC (19). In the works of many authors, mutations in the SDH complex—SDHD, SDHB, SDHC, SDHAF2—have been confirmed to be more frequent in multiple tumors (4, 12, 16, 20). When multiple tumors are found, they may be part of genetic syndromes associated with paraganglioma formation (1, 7, 13, 33, 35, 36). In contrast to multiple tumors, mutations in SDHB have been found in this group of patients when the ability to give metastasis to other organs such as lymph nodes, bone, liver (1, 11, 12). Due to the more malignant clinical course in such cases, Zhu et al. (34) authors suggested including family members in genetic and clinical care in situations where SDHB mutations were found. In the genetic diagnostic algorithm, Gupta (31) proposes separating the group of patients with multiple tumors into those with and without metastasis, and if metastatic features are found, perform first-line testing for SDHB. In light of the presented study results, it seems reasonable to include such a procedure in the genetic diagnostic algorithm. When planning diagnosis of family members can furthermore take into account the gender of the patients, which is important in determining the mutation carrier. Transmission of the mutation from the father increases the risk of behavior, while in the case of carrier in the mother, there is no need for screening in children (4, 16, 23).

Some controversy surrounds genetic testing in patients with isolated eardrum paraganglioma (Fisch A type). In a 2009 Burnichon study, 330 patients were studied with tumors in the tympanic cavity and none of them had mutations typical of HNPGL (12). However, the paper did not specify the number of patients with this type of paraganglioma. Based on the results, it was concluded that patients with isolated eardrum HNPGL did not require genetic testing. In subsequent studies, this group of patients was excluded from genetic diagnosis (12). In 2012, Piccini et al. (17) included these patients in their study and obtained positive mutation results in 6/16 patients, of which 3 patients with type A paraganglioma were accompanied by multiple lesions and 3 had isolated tumors of the tympanic cavity. Also, isolated eardrum HNPGL were diagnosed in a study by Heesterman (23), where patients with SDHD mutations were observed. Therefore, it seems reasonable to include patients with tympanic PGLs in future genetic studies, not only in situations where they are part of multifocal tumors.

In light of the findings presented regarding the necessity of genetic testing in HNPGL in patients with risk factors. There remains, however, a group of patients with isolated tumors not burdened by such factors. Previous publications have shown mutations in this group of patients as well, such as in the work of Mannelli (19) where 14.3% of patients with a negative family history and isolated tumors were found to have mutations. This study was extended several years later by Piccini (17) where mutations were found in 18.8% of patients without risk factors. Also among the patients studied by Chen et al. (22) were patients with SDHD mutations without risk factors. Therefore, a modern diagnostic algorithm should include all patients with HNPGL.

In the analyses presented so far, studies for the Polish population have been included in a multicenter study by Neumann et al. (20), who included 23 patients from Poland. However, the results were analyzed collectively for the entire group of 598 patients with HNPGL, so it is difficult to draw conclusions for our country’s population on this basis. In 2008, a description of 2 cases of familial HNPGL in Polish patients was presented (37). In 2015, a paper describing 14 patients with multiple head and neck chaperones was also published based on the Polish group (38). In none of these papers, however, genetic testing was performed, so it is reasonable to initiate genetic testing also in the Polish population in order for the management algorithm to be based not only on data from the literature but also on the results of the study.

## Conclusion

A genetic diagnosis of paraganglioma should be an integral part of the diagnostic process. The use of this diagnostic tool enables the early detection of pathological lesions and the initiation of treatment in family members of patients, thereby reducing the risk of complications. Furthermore, the rationalization of genetic diagnostics has an impact on the cost and efficiency of the process, which may lead to the applicability of diagnostic algorithms in clinical practice.

## Data availability statement

The original contributions presented in the study are included in the article/supplementary material, further inquiries can be directed to the corresponding author.

## Author contributions

KR: Conceptualization, Investigation, Methodology, Writing – original draft, Supervision. ZL: Data curation, Formal analysis, Visualization, Writing – original draft. RB: Supervision, Writing – review & editing. MZ-Ł: Project administration, Writing – review & editing. AR: Software, Writing – review & editing. JL: Writing – review & editing, Project administration, Software. MS: Supervision, Writing – review & editing.
